# Streamlining Systematic Reviews: Harnessing Large Language Models for Quality Assessment and Risk-of-Bias Evaluation

**DOI:** 10.7759/cureus.43023

**Published:** 2023-08-06

**Authors:** Abdulqadir J Nashwan, Jaber H Jaradat

**Affiliations:** 1 Nursing Department, Hamad Medical Corporation, Doha, QAT; 2 School of Medicine, Mutah University, Al Karak, JOR

**Keywords:** evidence synthesis, machine learning, artificial intelligence, large language models, risk of bias, quality assessment, systematic reviews

## Abstract

This editorial explores the innovative application of large language Models (LLMs) in conducting systematic reviews, specifically focusing on quality assessment and risk-of-bias evaluation. As integral components of systematic reviews, these tasks traditionally require extensive human effort, subjectivity, and time. Integrating LLMs can revolutionize this process, providing an objective, consistent, and rapid methodology for quality assessment and risk-of-bias evaluation. With their ability to comprehend context, predict semantic relationships, and extract relevant information, LLMs can effectively appraise study quality and risk of bias. However, careful consideration must be given to potential risks and limitations associated with over-reliance on machine learning models and inherent biases in training data. An optimal balance and combination between human expertise and automated LLM evaluation might offer the most effective approach to advance and streamline the field of evidence synthesis.

## Editorial

Large language models (LLMs) present a groundbreaking opportunity to revolutionize the process of conducting systematic reviews, particularly in quality assessment (QA) and risk-of-bias (ROB) appraisal. This lies in the potential of LLMs to automate the inherently labor-intensive and often subjective process of these tasks that may lead to inconsistencies among different assessors.

Systematic reviews conduct rigorous and comprehensive assessments of existing literature on a particular medical topic [1]. They are an essential part of evidence-based medicine and involve several stages, of which the QA of included studies or the appraisal of ROB is a critical stage to ensure the credibility of the review. These reviews critically analyzed the available evidence, identified potential biases or shortcomings in individual studies, and synthesized the findings [1].

LLMs are advanced artificial intelligence (AI) models, such as Generative Pre-trained Transformer 4 (GPT-4), DALL-E, Segment Anything Model (SAM), Large Language Model Meta AI (LLaMA), Language Model for Dialogue Applications (LaMDA), Vision Transformer, etc., capable of automating various tasks in natural language processing, computer vision, etc. GPT-4 is the latest GPT model incorporated in ChatGPT, which is a multimodal foundation model that excels in generating human-like text and can process text and image inputs and beyond [2]. LLMs are trained on extensive text data, enabling them to understand context, predict semantic relationships, and extract relevant information from diverse datasets [2]. Consequently, LLMs can be effectively harnessed to automatically assess the quality of the included studies in a systematic review, streamlining the process and reducing the subjectivity associated with human independent assessors.

Although QA and ROB assessments are considered similar safeguard elements and are sometimes used interchangeably by researchers, their applications and interpretations differ. QA evaluates methodological safeguards within a study to ensure it is well-designed, conducted, analyzed, interpreted, and reported, thereby minimizing systematic errors or bias. Conversely, the ROB assessment, also known as critical appraisal, aims to understand how these safeguards may influence the study results, involving judgments about the level of bias and delving deeper into the implications of methodological safeguards on the study's outcomes. QA entails counting the safeguards present as numerical assessment, whereas a ROB assessment employs an approach to rank studies based on potential bias into low or high-risk studies [3,4]. There are numerous tools to assess QA and ROB; they differ according to the study design, such as the Joanna Briggs Institute (JBI), Assessing the Methodological Quality of Systematic Reviews (AMSTAR), Critical Appraisal Skills Program (CASP), Cochrane Risk of Bias (ROB 1 and 2) tool, Risk Of Bias In Non-randomized Studies - of Interventions (ROBINS-I) tool, the Newcastle-Ottawa Scale (NOS), etc. [4].

LLMs can be trained to identify common sources of bias, such as selection, performance, detection, attrition, and reporting, by recognizing specific phrases, language patterns, or missing information; the LLM can assign a risk level of bias to each study. This objective, automated assessment can minimize human assessors' subjectivity, enhance the systematic review's reliability and capture nuances that may otherwise have been overlooked [5]. The use of LLMs not only improves the efficiency and consistency of QA and ROB evaluations but also expedites the overall systematic review process. By eliminating the need for manual appraisal, LLMs enable faster evidence synthesis and knowledge dissemination, crucial in developing clinical guidelines and public health emergencies (e.g., pandemics), where timely access to highly-quality synthesized evidence is crucial. However, using LLMs holds many potential risks, limitations, and challenges. Overreliance on LLMs may weaken critical thinking skills in researchers. Risks associated with inherent biases in the training data of LLMs might influence the assessment outcomes [2,5]. Therefore, combining human expertise and automated LLM evaluation might offer the best approach, ensuring both the efficiency of automation and the nuanced understanding of human assessors.

In conclusion, harnessing LLMs in conducting quality assessments and the risk of bias in systematic reviews can be transformative. With proper caution to ensure appropriate use and mitigate potential risks, efficiency, consistency, and objectivity benefits can significantly advance the evidence synthesis field.
